# How to deliver person-centred care for people living with heart failure: a multi stakeholder interview study with patients, caregivers and healthcare professionals in Thailand

**DOI:** 10.1186/s12913-024-11922-z

**Published:** 2024-12-18

**Authors:** Alessandra Giusti, Panate Pukrittayakamee, Kamonporn Wannarit, Lakkana Thongchot, Satit Janwanishstaporn, Kennedy Nkhoma, Sridhar Venkatapuram, Richard Harding

**Affiliations:** 1 University of Cambridge, The Healthcare Improvement Studies Institute, Cambridge, UK; 2 King’s College London, Cicely Saunders Institute of Palliative Care, Policy and Rehabilitation, London, UK; 3 Mahidol University, Faculty of Medicine, Siriraj Hospital, Bangkok, Thailand; 4 University of Johanessburg, Johannesburg, South Africa; 5 King’s College London, King’s Global Health Institute, London, UK

**Keywords:** Health Services Research, Person-Centered Care / Person-Centred Care, Patient-Centered Care/ Patient-Centred Care, Qualitative Research, Empirical Research, Heart Failure

## Abstract

**Context:**

Heart failure has high, growing global prevalence, morbidity and mortality, and is a leading cause of death with serious health-related suffering in low- and middle-income countries. Person-centred care (PCC) is a critical component of high-quality healthcare and is particularly vital in the context of a serious illness such as heart failure. However, there are limited data exploring PCC in this population in low- and middle-income settings.

**Aim:**

The aim of this study was to explore how clinical services could respond to the PCC needs of individuals living with heart failure in Thailand, with potential for adaptation in other settings. The specific objectives were (i) to understand the experiences and needs of persons living with heart failure, their caregivers and HCPs; (ii) to explore specific practical actions that can help deliver PCC for heart failure patients in this setting.

**Methods:**

Cross-sectional qualitative study. In depth, semi-structured interviews were conducted in Thailand with heart failure patients (*n* = 14), their caregivers (*n* = 10) and healthcare professionals (*n* = 12). Framework analysis was conducted with deductive coding to populate an a priori coding frame based on Santana et al’s PCC model (2018) and Giusti et al’s systematic review (2020), with further inductive coding of novel findings to expand the frame. The study is reported in accordance with the consolidated criteria for reporting qualitative research guidelines (COREQ).

**Results:**

The findings reveal specific practice actions that deliver PCC for persons living with heart failure in Thailand, such as (i) compassionate communication by healthcare professionals; (ii) effective teamwork amongst multidisciplinary healthcare professionals; (iii) proactive responses to physical, psychosocial, relational and information needs of patients and caregivers; (iv) engaging patients and families in symptom management; (v) providing opportunities for patients to be cared for in the community; and (vi) responding to the social determinants of health, illness and healthcare access.

**Conclusion:**

Person-centred healthcare systems must aim to address the social determinants of illness and place focus on community- and home-based care. Heart failure patients and caregivers must be supported to self-manage, including how to recognise symptoms and take appropriate action. Delivering PCC in such a way has the potential to improve outcomes for patients, enhance patients’ sense of agency and experiences of care, improve health equity, and reduce hospital admissions, relieving pressure on the hospital system and reducing overall costs of care.

**Supplementary Information:**

The online version contains supplementary material available at 10.1186/s12913-024-11922-z.

## Key message

This multi stakeholder study reveals specific practical actions that can contribute towards delivering person-centred care for persons living with heart failure in Thailand, with potential for adaptation in other settings.

**Supplementary Information:**

The online version contains supplementary material available at 10.1186/s12913-024-11922-z.

## Introduction

Heart failure is a serious, progressive physical illness, affecting more than 64 million people globally [1, 2]. In low- and middle- income countries (LMICs), heart failure is one of the leading causes of death with serious health-related suffering [3]. Unlike most cardiovascular diseases, the global incidence of heart failure is rising rapidly [1–4], and it is now a dominant form of cardiovascular disease in South East Asia and a leading cause of death in Thailand [5–7]. Individuals living with heart failure have multidimensional needs [8–10], typically experience a high burden of physical and psychological symptoms, and report reduced quality of life [11, 12]; Symptoms such as pain often remain under-recognised and undertreated [13, 14]. Heart failure is further associated with unpredictability in symptom exacerbation, frequent use of health services, and unplanned and prolonged hospital admissions [15, 16]. This serious, life-threatening and life-limiting illness therefore places a considerable burden on society, health systems, individuals with heart failure, and their families.

In the context of a serious illness such as heart failure, person-centred care (PCC) is especially critical. The complex clinical scenarios caused by heart failure usually demand high-quality communication, management of clinical uncertainty, the involvement of family members, and joint decision-making to deliver care aligned with patient preferences and multidimensional needs [17–20]. PCC for serious illness has the potential to provide multiple benefits for patients, families, staff and the healthcare system in terms of engagement, enablement, adherence to treatment [21], patient satisfaction [22], management of symptoms [23], reduction in re-referrals [9], and improved patient health outcomes [24–26].

A variety of terms have been used to denote person-centred approaches, including ‘patient-centredness’ and ‘people-centredness’. Still, person-, patient- and people-centred care all embody an approach that adopts the perspectives of individuals, families and communities, respects and responds to their needs, values and preferences, and sees them as active participants in their own healthcare [27–29].

The concept of person-centred care is now established within global health policy dialogue. Since the US National Academy of Medicine listed PCC as one the six aims for healthcare improvement [27], there has been a paradigm shift from paternalistic, disease-focused policy models towards humanised, holistic PCC. National governments [30–32], international organisations such as the World Health Organization [33], and patient and health policy groups [34–39] emphasise the need for healthcare to be person-centred.

The WHO global strategy on people-centred and integrated health services acknowledges that PCC should be context-specific and that each country should generate local evidence to enable appropriate, feasible practice of PCC [28]. However, ‘person-centred care’ is a concept that has been conceptualised within a few high-income countries and limited data exists to model contextually and culturally appropriate PCC in LMICs [40, 41]. There is little evidence on precisely how person-centeredness should be understood and delivered for heart failure patients and their families in LMICs and in Thailand specifically. A recent systematic review of the existing evidence underpinning conceptualisations of ‘person-centredness’ for serious illness [40] did not identify any empirical studies originating from LMICs or Southeast Asia, and did not identify any studies focusing on heart failure.

In Thailand, universal health coverage has been provided since 2002 through three programs: the Civil Servants’ Medical Benefits Scheme for civil servants and their families, the Social Security scheme for private employees, and the Universal Health Coverage (UHC) scheme which is available to all other Thai citizens and covers around 75% of the population. The UHC scheme ensures that the entire Thai population has health insurance, including low-income households, and enables patients to access services in their health district and, if necessary, to be referred for specialist treatment elsewhere [42–44]. Elements of PCC, as conceptualised in high-income countries, have been promoted in Thailand for several decades [45]. In 1991, leaders across a range of primary health centres began demonstrating new approaches to care, including regular community meetings to hear the public’s views and emphasising privacy, listening, and discussion during clinical encounters [45]. According to the WHO, “following Thailand’s universal coverage reforms of 2001, this model of care was adopted by the government as the cornerstone of its new primary care-based health care system” (p8) [45]. However, it is not clear in what ways, and to what extent, clinical services in Thailand are addressing the person-centred care needs of persons living with heart failure and their families.

The aim of this study was to explore how clinical services could respond to the needs of individuals living with heart failure in Thailand, with potential for adaptation in other settings. The specific objectives were (1) to understand the experiences and needs of persons living with heart failure, their caregivers and HCPs; (2) to explore specific practical actions that can help deliver PCC for heart failure patients in this setting.

## Methods

### Design

This cross-sectional, qualitative study used semi-structured interviews and framework analysis [46]. It is reported in accordance with the consolidated criteria for reporting qualitative research guidelines (COREQ) [47] (see Supplementary File 1).

### Setting

Participants were recruited from the cardiology outpatient unit in a large public teaching hospital in Bangkok, Thailand. The heart failure clinic provides multidisciplinary outpatient healthcare services to ACCF/AHA stage C or D heart failure patients.

### Sampling and recruitment

The study sampled patients, informal caregivers and healthcare professionals (HCPs). Clinicians at the site approached patient and caregiver participants regarding the study in person. Those who expressed interest in participation were provided with more detailed information by a local research assistant. Eligible HCPs were introduced to the study by their facility manager, who provided an information sheet and contact details for the local study research assistant. Written consent was obtained from all participants. The study team were careful to ensure that an ethical approach to consent was taken; Actions taken include providing prospective participants with culturally and language appropriate consent materials, and ensuring they were given the opportunity to discuss any risks and benefits of participants in detail with the local research assistant.

Inclusion criteria for patient participants: aged 18 years or over; having physical and psychological capacity to consent and participate; receiving care from the study site; and diagnosed by their treating clinician as having with ACCF/AHA stage C or D heart failure. Inclusion criteria for informal caregivers: aged at least 18 years; were caring for someone who met the criteria for patients and who was being cared for at the recruitment site; and met the definition of “*unpaid*,* informal providers of one or more physical*,* social*,* practical and emotional tasks. In terms of their relationship to the patient*,* they may be a friend*,* partner*,* ex-partner*,* sibling*,* parent*,* child or other blood or non-blood relative*” [48]. Inclusion criteria for HCPs were any healthcare staff who had worked at the study site clinically providing direct care to heart failure patients for at least 6 months. For all populations sampled, participants were required to be able to communicate in either Thai or English.

Patients were sampled purposively to achieve heterogeneity with respect to age, gender, stage of heart failure, socioeconomic status and primary concurrent treatment. Caregiver participants were purposively sampled by age, gender and relationship to patient, and HCPs were purposively sampled by age, gender, professional role and years of experience. Cases were sought that represent a wide variety of experiences, expectations, needs and opinions, enabling a more comprehensive and nuanced understanding of person-centred care needs in the context of heart failure.

### Data collection

In-depth semi-structured interviews, along with demographic data collection, were conducted face-to-face at the study site during routine outpatient visits. Participants were also given the option of participating by telephone due to COVID-19 restrictions. Data were collected between July 2020 and April 2021.

All interviews were conducted by PP, a male practicing psychiatrist with an MSc in Palliative Care. None of the participants were previously known to PP, although all were informed that he was a practicing psychiatrist with an interest in improving patient care. The interviewer participated in two qualitative research training workshops led by AG. Semi-structured topic guides were used to conduct the interviews. Interviews with patients and caregivers comprised open questions focusing on the interviewee’s main needs, values, priorities and concerns, whether/how those needs were being met, how they would describe the care received, and how they would want care to be delivered. The topic guide was adapted for HCPs to include questions focusing on current practice, their views on anticipated challenges and benefits of reorienting practice to a person-centred approach, and their training and support needs. (Supplementary Material 3 further details the content and development of these topic guides).

Data collection continued until it was collaboratively decided by PP and AG that sufficient information power had been reached [49]. In line with Malterud et al.’s (2016) concept of information power, five factors were considered in determining when sufficient information power would be reached: the broadness of the study aim, the characteristics of the sample, the existence of relevant theory, the quality of the interview dialogue, and the planned analysis method. Based on these factors and the researchers’ experience, it was anticipated that sufficient information power would be reached once approximately 20 patients, 15 informal caregivers and 15 healthcare professionals had been interviewed.

Interviews were digitally audio recorded, transcribed verbatim by the interviewer, translated into English, and pseudonymised. *N* = 18 interviews were translated by a bilingual colleague of the interviewer and *n* = 18 were translated by an external service (See Supplementary Material 4 for further details of transcription and translation process). A ‘reflexivity log’ (Supplementary Material 5) was completed following each interview to record contextual factors, emergent themes, reflections and inform assessment of information power and the analysis process.

### Analysis

Data were analysed using framework analysis [46], combining deductive and inductive approaches, and using NVivoPro software to manage the data. Santana et al.’s PCC model [50] and Giusti et al.’s review [40] of data underpinning the concept of PCC for serious illness were used to construct an a priori coding frame (Supplementary figure A) for deductive data analysis, with additional inductive coding for data that further expanded the a priori frame. The PCC model developed by Santana and colleagues [50] was selected to create a priori codes as it provides comprehensive, practical guidance for implementation of PCC, explicitly linking this guidance to the Donabedian model [51] for assessing healthcare quality. The coding framework was constructed collaboratively, drawing on the local researcher’s knowledge and views throughout. First, the lead researcher in Bangkok (PP) participated in a qualitative data analysis workshop led by AG. PP and AG both then individually coded a sub-selection of the interview transcripts: three patient, three caregiver and three HCP interviews. PP and AG then reconvened to compare coding and develop a coding frame; PP and AG discussed all codes in depth, coming to a consensus and shared meaning for each code. KN and RH were consulted to resolve any differences of opinion. Whilst coding and developing the coding frame, the researchers referred back to the reflexivity log for each interview to ensure that their interpretations considered contextual and non-verbal information from each interview and accounted for any key insights and themes documented by the interviewer.

The coding frame that developed comprised: preselected a priori codes consisting of Santana et al. PCC model domains [50], a priori codes derived from the results of a previously conducted systematic review [40], and inductive codes derived by content-related open coding. AG then coded all the remaining transcripts using the agreed coding frame. AG indexed and sorted all interview transcripts, created a framework matrix for each broad coding frame category, and led mapping and interpretation of the data. Key findings were mapped into a framework of PCC and organised by WHO building blocks for strengthening health systems [48].

This collaborative process for data analysis enhanced dependability, strengthened the analysis and resulting findings. Ideas, hypotheses and decisions were noted in NVivo memos throughout the analysis process to serve as an audit trail and enhance confirmability.

Member checking was conducted during a face-to-face workshop in May 2024 with a selection of HCP and patient participants. Patient and public involvement was not conducted as part of this study.

The data from this study was also analysed as part of a broader study investigating the meaning and practice of PCC. The broader study aimed to construct a practice-based framework to strengthen health systems through PCC based on novel primary data [29].

### Research governance

Ethical approval was granted for the study by King’s College London Research Ethics Committee (HR-19/20–14952) and by Siriraj Institutional Review Board, Faculty of Medicine Siriraj Hospital, Mahidol University (Si 652/2020).

All study data were collected, handled, and stored in full compliance with the UK Data Protection Act 2018 (General Data Protection Regulation (GDPR)).

## Results

### Participants

We recruited *N* = 36 participants (see Table 1): *n* = 14 heart failure patients, *n* = 10 caregivers and *n* = 12 HPCs. As these interviews yielded rich, high quality data that addressed the study aims, and as reflexivity logs indicated recurrent themes amongst later interviews, it was deemed that sufficient information power [44] had been reached for meaningful conclusions to be drawn. Purposive sampling parameters were achieved (see Table 1). The mean duration of interviews was 47 min (range: 22–83 min). The recruitment rate was 26%. *N* = 35/36 interviews were conducted face-to-face in the health facility and *n* = 1/36 interview was conducted by telephone (participant ID 1014).

**Table 1:** Participant characteristics (*n* = 36)

Patient participants	*N* = 14	Caregiver participants	*N* = 10	HCP participants	*N* = 12
**Gender** (Male/Female)	11/3	**Gender** (Male/Female)	1/9	**Gender** (Male/Female)	1/11
**Age** (years)		**Age** (years)		**Age** (years)	
Mean (SD)	54 (14.8)	Mean (SD)	50.4 (8.2)	Mean (SD)	28.7 (8.5)
Range	22–81	Range	35–62	Range	20–43
**Education level**		**Education level**		**Professional role**	
Primary school	2	Junior high school	1	Nurse	7
Junior high school	1	Senior high school	1	Practical nurse	5
Senior high school	1	Bachelor’s degree	5		
Bachelor’s degree	6	Higher than bachelor’s degree	2	**Years of experience as HCP**	
Higher than bachelor’s degree	2	Vocational diploma	1	Mean average (SD)	6.9 (8.1)
Vocational certificate /Diploma	2			Range	0.5–21
**Occupation**		**Occupation**		**Years of experience working with heart failure patients**	
No occupation	8	Employee	2	Mean average (SD)	5.6 (7.1)
Government employee	3	No occupation	2	Range	0.5–21
University employee	1	Self-employed	4		
Merchant	2	Government employee	1		
		Salesperson	1		
**Marital status**		**Marital status**			
Single	3	Single	3		
Married	8	Married	6		
Divorced	2	Divorced	1		
Widow	1				
**Religion**		**Relation to patient**			
Buddhism	14	Spouse	3		
		Son/Daughter	6		
		Parent	1		
		**Years of caring for patient**			
		Mean average (SD)	7.5 (6.9)10		
		**Religion**			
		Buddhism	10		

*SD* Standard deviation, *HCP* Healthcare professionals

### Findings

The findings are grouped by PCC components proposed in the 2022 practice-based framework of PCC [29]; In this framework [29], components are presented as structures, processes and outcomes of PCC, and organised by WHO building blocks for strengthening health systems. The findings from this study fall within PCC components related to three WHO health system building blocks: (i) health workforce (ii) service delivery and (iii) health information systems.

### Health workforce

#### Values and attitudes

Patients and caregivers often reported the importance of HCPs having positive and caring attitudes for their emotional wellbeing and experience of a healthcare facility. In particular, participants commonly highlighted the importance of HCPs being non-judgemental and not placing blame on the patient (quotes 1, 2, see Table 2). Participants further suggested that HCPs should treat patients and caregivers with equal respect and urgency, regardless of the service user’s financial and social status (quote 3).

#### Teamwork

A dominant view amongst HCPs was the need to promote a supportive work environment for the health workforce that encourages effective and harmonious multidisciplinary teamwork working and, ideally, development of amicable relationships to promote understanding and collaboration (quote 4). HCPs discussed the importance of supportive relationships at work and the value of being able to debrief and seek advice from colleagues (quote 5).

#### Communication skills

Patients, caregivers and HCPs all stressed the importance of clinicians’ communication skills. In particular, HCPs identified the need for training in supporting patients and families psychologically and in sensitive communication (quotes 6).

Patients and caregivers described the value of feeling heard and described listening as a way of HCPs showing respect, building trust and building a patient’s confidence. Patients expressed a need to be listened to both in terms of their immediate physical needs, such as pain relief or side effects, and in terms of their broader life circumstances and concerns (quote 7). Patients also wanted HCPs to encourage them to raise topics, ask questions freely, seek clarifications and express their views and concerns, whilst being made to feel comfortable in doing so (quote 8).

Participants across all stakeholder groups highlighted the need for HCPs to communicate and share information in ways that patients would understand. This included using non-medicalised, layman language, being sure to repeat important information, and making particular effort to check understanding for patients and families communicating in a language that is not their first (quote 9).

#### Wellbeing

The health workforce were reported as often being overstretched (quote 10). Participants also reported the psychological challenges of caring for patients who were seriously unwell or dying and suggested the need for psychological support for HCPs. Some HCPs described coping strategies they used to cope with the emotional toll of their profession, such as debriefing with co-workers (quote 11) (Table 2).

**Table 2:** Illustrative participant quotations for Health Workforce building block

**Values and attitudes**	
*1*	*“All doctors here are very kind. I feel relaxed when I talk to them. They never scold me. This makes me want to engage with the treatment. They encourage me so I don’t feel down. The previous hospital did not make me feel this way. I used to be against treatment since my symptoms did not get better and nurses scolded me.” 1006, Patient, Female, 58*
*2*	*“When my symptoms get worse, I will tell the doctor what I have done…When I have been drinking [alcohol], I tell the doctor so that he has information to decide on the treatment.” 1009, Patient, Male, 38*
*3*	*“[The doctor in another hospital] told us that he could not treat the patient’s symptoms…We also felt so bad and thought that the doctor might want to treat someone richer than us. That was my feeling.” 2006, Caregiver, Female, 62*
**Teamwork**	
*4*	*“We have collaborated for a long time; we get along well including the doctor, nurse, and pharmacist and we understand each other. Having a personal bond helps us collaborate well, that is, our communication runs smoothly, and we work with no pressure.” 3010, HCP, Female, 43*
*5*	*“We can consult other specialties when we have problems. We can also communicate with a doctor or a pharmacist directly. We can ask a doctor when we need more information or would like to know his opinion regarding patient’s symptoms.” 3002, HCP, Female, 24*
**Communication skills**	
*6*	*“I think we need an intensive training because talking to patients in the final stage requires advanced communication skill. One wrong word can change everything. If I made them feel saddened, I would feel guilty for a long time.” ID3007, HCP, Female, 27*
*7*	*“I think that talking is good for me, as in asking about my living and suggestions on personal practice, this would give me courage. Asking about my life will make me feel at home.” 1007, Patient, Male, 60*
*8*	*“I like the doctor gives me opportunities to ask questions.” 1014, Patient, Male, 41*
*9*	*“I’d like him to use simple language not medical terms. I can never remember the name of medicines since their names are unfamiliar…I also need simple explanations on how organs work. Using medical terms makes me confused.” 1005, Patient, Male, 52*
**Wellbeing**	
*10*	*“I do understand that doctors there deal with a lot of patients and nurses see patients every day, this can cause them stress.” ID 1007, Patient, Male, 60*
*11*	*“I think some staff may need to see a psychiatrist in order to vent their feelings so that they can smile when they see patients. I wish they could look back and see that their works can be very useful for patients and how much they can help other people. If they are aware of these, they might be more friendly to patients. But if they don’t have a chance to cope with their feelings, they may only focus on their workload.” ID 2005, Caregiver, Female, 50*

### Service delivery

#### Responsive to needs, preferences and values

Patients, caregivers and HCPs stressed the importance of services proactively responding to patient’s preferences and changing needs, particularly emotional or psychological needs, as well as physical health needs (quote 12). Participants reported high prevalence of anxiety, fear, irritability, and/or depression amongst persons living with heart failure; Fear of experiencing cardiac arrest and fear of heart surgery was commonly shared (quotes 13). Participants described the importance of HCPs identifying, acknowledging and taking time to address these needs, for example by referrals to specialist support or teaching self-management techniques to encourage a sense of control. Heart failure was also often reported to have significant impacts on the psychological health of informal caregiver(s) or close family member(s), including causing severe stress, worry and sleeplessness (quote 14); This highlights the importance of considering and addressing caregivers’ psychological needs, in addition to those of the patient.

Participants also described examples of social needs, such as financial challenges or unemployment, and some explained ways in which these needs were addressed through service delivery, such as by signposting the patient to available financial assistance funds (quote 15). These social needs were experienced by caregivers, as well as patients, who in some instances had taken time away from work or education to care for the patient or accompany them to appointments, and faced the financial consequences of doing so (quote 16).

Participants reported the potentially significant impact of heart failure on a person’s ability to carry out their normal roles and daily tasks, and on the person’s future plans and life aspirations (quote 17). Participants suggested the need to support patients with participating in regular personal life activities, including socialising, hobbies, and employment (quote 18). A consistent view was the importance of social interactions and relationships to human wellbeing. Participants often described social isolation as a negative impact of living with heart failure. Some patients reported enjoying the social interactions that came with frequently attending a healthcare facility or receiving home visits (quotes 19, 20).

Participants also frequently spoke of patients’ and caregivers’ information needs. The dominant view of both patients and caregivers was that they should be given detailed, honest information about the condition and treatment options in order to better understand why a particular treatment plan would be best suited to them and how their life would be affected. Caregivers suggested that receiving information about the person’s diagnosis, symptoms and medication side effects enabled them to feel more confident in caring for the person and reduce feelings of helplessness (quotes 21, 22). Participants also described the need for frequently monitoring disease progression and discussing this in detail with the patient, educating the patient about ways in which their condition is influenced, and encouraging lifestyle behaviours to improve health status (quotes 23). A widespread view across all stakeholder groups was that a patient’s diagnosis, disease stage and prognosis must be shared honestly, yet gently and gradually. Participants stressed the importance of communicating with sensitivity and carefully judging how and when sensitive topics should be discussed with patients and their family members depending on the individual patient’s personality and psychological health (quote 24).

Conversely, some patients and caregivers expressed a preference for not knowing the future treatment plan and being informed of each upcoming step only. Underlying this preference was often a fear of increased anxiety about the future, a focus on relief from pain, or a desire to ‘live life day-by-day’ (quote 25). HCPs also noted a self-tendency to only discuss immediate next steps for fear their patients would become discouraged and worried about the future (quote 26).

#### Community-oriented services

Patients, caregivers and HCPs stressed the need to provide opportunities for patients to be cared for in the community and at home, through home visits and equipping the home for their needs (quote 27). Providing home visits and preventing patients from having to travel to facilities was further seen to prevent exhausting unwell individuals and improve care access for those living far from facilities (quote 28). Participants viewed home visits as helpful for delivering home equipment and medications, setting up a health-promoting environment and upskilling the patient and household in self-management (quote 29, 30).

To deliver services and care in the home and community, participants suggested drawing on human resources from the community, upskilling persons such as health promoters and volunteers, and developing strong coordinated linkages with voluntary and specialist services in the community (quotes 31, 32).

Another widespread view was the value of condition-specific group-based patient support (quotes 33). This was seen as enabling peer learning and allowing persons living with heart failure to empathise with and support each other. Group activities were also viewed as enabling social interactions and reducing social isolation.

#### Social determinants of health

Participants often reported how social conditions can contribute towards a person’s health or ill-health (quote 34, 35). Participants pointed towards a need to consider and address the social determinants of health, including housing conditions, employment, education and financial status. Participants alluded to ways in which material or social situations can constrain a person’s ability to engage with health services or their care or adhere to treatment. For example, participants described an inability to continue with a treatment plan due to high costs (quote 36).

#### Engaging patients and caregivers in managing their care

Patients commonly expressed a desire to take more control over their own health, requiring education in using home monitoring equipment and wider self-management education to manage their condition at home (quote 37). HCPs strongly supported this need for health education (quote 38). Participants also raised the importance of providing patients and caregivers with appropriate education, support, sufficient medication and equipment, such as blood pressure monitors and oximeters, to enable self-care and self-management of symptoms (quote 39).

#### Care integration and coordination

Participants highlighted the importance of streamlining and easing patient navigation, ensuring continuity of care and simplifying the process of multi-specialist care. Participants suggested the need to simplify care pathways through approaches such as: establishing clear points of contact or care access; providing a ‘one-stop-shop’ service where possible; building smooth and swift referral pathways; and easing the process to transfer health financial coverage across facilities (quote 40, 41).

Others described coordinated information sharing between all healthcare professionals and specialist providers along a patient care pathway (quote 42). Described methods of ensuring coordinated information sharing included: face-to-face interdisciplinary meetings, an accessible care coordinator, detailed handover communication between HCPs at shift changes, inter-departmental online communication systems, and improved accessibility to digital medical information (quote 43) (Table 3).

**Table 3:** Illustrative participant quotations for Service Delivery building block

**Responsive to needs, preferences and values**	
*12*	*“There should be an assessment of individual patient’s specific needs because individual patients have different needs.” 3008, HCP, Female, 42*
*13*	*“Mental health care is yet be taken seriously…. Most patients are worried about cardiac arrest.” 3012, HCP, Female*
*14*	*“Her illness has greatly affected me as I’m stressed and sleepless; I have to take a sleeping pill every night.” 2010, Caregiver, Female, 52*
*15*	*“Needy patients generally have difficulty with travel costs, so we send them to the department of social work for financial assistance…They were happy about it; we assisted them to the best of our abilities. We booked appointments for them as much as necessary.” 3021, HCP, Female, 20*
*16*	*“Since the patient got sick, I haven’t sold any goods; it’s been two years now”. 2010, Caregiver, Female, 52, Thailand*
*17*	*“She wants to pursue her study for two more years; now that she’s fallen ill, she complains every day that she wants to study. This makes her stressed, sad, and sleepless.” 2010, Caregiver, Female, 52*
*18*	*“We have to prepare an oxygen tank in our car when we have to go on a trip and we also have an electric oxygen machine in the bedroom since she likes to travel a lot.” 2001, Caregiver, Male, 52*
*19*	*“I live alone so talking to a doctor makes me feel like I am letting my friend know my problems, especially when I feel tired of my life. When my girlfriend left me, I talked to a pharmacist and cried with her.” 1001, Patient, Male, 44*
*20*	*“There’s an elderly lady who told me that she enjoyed the company of the doctor and nurse on their home visit.” 3008, HCP, Female, 42*
*21*	*“I want to know in detail what effects the medicines have. Normally, he tells us how each medicine works such as decrease hypertension, decrease body swelling and prevent blood clot. But I don’t know whether it is necessary to take this large number of medicines or not. I give the medicines to my dad without knowing the details. I really want to know if all medicines he is taking are necessary.” 2005, Caregiver, Female, 50*
*22*	*I like that the doctor directly explains the patient’s condition and the treatments to me. The doctor doesn’t keep the information to himself. That way, I can be prepared to do what I need to in order to handle the situation. 2003, Caregiver, Female, 43,*
*23*	*“The doctor should tell the patient the truth, informing them how future symptoms and treatment plans will be so that the patient can make a plan to adjust themselves. If I were a patient, I definitely wouldn’t want the doctor to keep it to themselves.” 3012, HCP, Female, 30*
*24*	*“I have to evaluate each patient. I have to know how much each of them can accept. If they seem to take it very well, I will let them know as much as they want to know. But if they are not ready to know, I will have to carefully communicate with them and help them to accept the information.” 3004, HCP, Female, 25*
*25*	*“I don’t want to talk about a long-term plan. I just live my life day by day.” 1001, Patient, Male, 44*
*26*	*“Usually, doctors tell patients only about their current condition because if they talk about the future, patients would be discouraged and wouldn’t want to continue the treatment.” 3011, HCP, Female, 25*
**Community-oriented services**	
*27*	*“A home visit would be great. We would feel that the team and the hospital care for us. It would encourage us to go on.” 2005, Caregiver, Female, 50*
*28*	*“Home visits will help us better follow up with patients’ symptoms. Relatives of bedridden patients will have trouble bringing the patients to the hospital. If we can pay a home visit, it would be convenient for them.” 3009, HCP, Female, 21*
*29*	*“The hospital’s home visit team…They would basically observe the patient’s living conditions such as residence and environment and would provide any equipment, if needed. They also gave guidelines on personal care at home.” 3008, HCP, Female, 42*
*30*	*“I would like them to give some advice on home arrangement. We’d like them to see whether the toilet is appropriate for the patient or not. If they see our house, they may give some further relevant advice, especially how we can prevent her from falling and how to maintain cleanliness and hygiene.” 2002, Caregiver, Female, 53*
*31*	*“A village volunteer’s visit would be nice to help patients. If the patient’s condition worsens, there would be someone to inform the relatives to take the patient to the hospital.” 1010, Patient, Female, 60*
*32*	*“It would be good if we can have more home visits and more collaboration with local hospitals. We already advise patients who don’t have a blood pressure meter at home to let a volunteer villager help them monitor their blood pressure.” *3002, HCP, Female, 24
*33*	*“It is very necessary to talk about this with other patients who have medical conditions. It would be good if we could talk to someone with knowledge. I used to watch a TV program that let the elderly talk to one another about their health. I could use the knowledge I got from their experiences to take care of myself.” *1004, Patient, Male, 81
**Social determinants of health**	
*34﻿*	*“Heart failure usually occurs to the needy; this makes them stressed about expenses. These patients generally have no relatives to look after them thus get neglected. Those who live by themselves will therefore become stressed; this makes their condition become aggravated.” *3010, HCP, Female, 43
*35﻿*	*“We should know about personal information that might affect patients’ condition such as their jobs because certain jobs can trigger a relapse.” *3009, HCP, Female, 21
*36*	*Some patients have to take medicines that are not covered by the Universal Health Coverage Scheme. Some of them used to ask us whether they could stop taking that medicine or not because it was so expensive.”* 3006, HCP, Male, 24
**Engaging patients and caregivers in managing their care**	
*37*	*“The doctor allows me to adjust Lasix intake by following the instructions advised on the adjustment...The fact that I could adjust my medication enabled me to look after myself at home. If my condition was to get a bit worse, I would increase the dose. If I urinated too much, I would lower the dose.” *1007, Patient, Male, 60
*38*	*“I think when they know how to cope with their symptoms and how to solve the problems, they may feel relieved from stress.” *3001, HCP, Female, 26
*39*	*“I think we need to be aware of how patients can take care of themselves at home. We normally...ask them to monitor their own weight at home. However, some patients do not have a weighing machine at home and some patients don’t know how to measure the amount of urine...Some of them don’t have a blood pressure monitoring device. It would be great if we could have these devices for patients to borrow.”* 3004, HCP, Female, 25
**Care integration and coordination**	
*40*	*“Like today after I finished my meeting with the doctor, they let me know where I should go next…They can communicate clearly. Sometimes when we go to a government office, we may not know who to contact and what to do. But here everything is very clear.”* 1003, Patient, Male, 54
*41*	*“If patients can access health care coverage at another hospital, we would advise the patients on how to transfer the coverage from the affiliated hospital to here. The affiliated hospital would allow a transfer of coverage for three months. After three months, we have to write up a document for the patient to bring to the affiliated hospital so that the hospital transfers the coverage to here…The affiliated hospital should allow for more than three months of health care coverage.”* 3009, HCP, Female, 21
*42*	*“They work together very well and step-by step. We have met a nurse, a doctor and a pharmacist and we have found that they transfer patient’s information among their team very well. They don’t ask the same questions.”* 2007, Caregiver, Female, 62
*43*	*“Before we refer to another unit, we contact that unit to inform them about our patient’s condition and other relevant information. We also advise our patient on the process and where they should contact. We will then scan patient’s information and send it to that unit. We can track whether our patient really goes to that unit and gets treatment or not on our computer.”* 3004, HCP, Female, 25

#### Health information systems

Participants discussed the value of electronic medical records and of developing or using e-health platforms for information exchange across healthcare providers and patients. Such infrastructure was deemed to improve accessibility of healthcare, information access, communication between patient and HCP and capacity for self-management (quotes 44–46). Nevertheless, a minority of patients raised concerns about reliance on technological communication, suggesting the need to consider inclusivity for those with lower technological skills (quote 47) (Table 4).

**Table 4:** Illustrative participant quotations for Health Information Systems building block

*44*	*“I’d like to be able to use technology to send information about my symptoms and blood pressure to the hospital so the doctor can recommend what I should do.” 1008, Patient, Male, 63*
*45*	*“Normally, a doctor will order us to do telemonitoring with patients after they visit our clinic… Patients will take a picture of their records and send it to us via the official LINE of the heart failure clinic.” 3004, HCP, Female, 25*
*46*	*“We used to bring the patient’s file to another hospital, but the handwriting was difficult, and some was in English. The doctor could not understand and had to ask many questions for medical history, but we could not remember it all. I believe if the medical history could be stored in a thumb drive which we can bring with us anywhere, that would be helpful.” 2009, Caregiver, Female, 45*
*47*	*“I’m not good at communicating via the Internet like this clinic does as I’m old- school and not good with computers.” 1007, Patient, Male, 60*

## Discussion

The study findings inform specific ways in which service delivery should respond to the PCC needs of heart failure patients. Firstly, the results indicate that HCPs recognise the benefits and necessity of community- and home-based care for heart failure patients. Community-oriented service delivery is especially important for persons living with heart failure, considering the long-term nature of the condition and the common exacerbations and unplanned hospital admissions. Service provision at decentralised levels of healthcare has been associated with improved health outcomes [52, 53], although in Thailand (and most LMICs) accessible services close to people’s homes are not available [54, 55]. The study findings suggest that accessible, community-based care may be enhanced by providing home visits to advise heart failure patients and their families on symptom control and health behaviours, and by building links with existing ‘assets’ and human resources in the community, such as community volunteers, social workers and local groups. Community health workers must be managed in close coordination with the facility workforce and other services, and specific attention and resources must be directed towards training, supporting, and compensating this cadre of workers [56, 57].

Secondly, the findings highlight that service delivery must respond to the social determinants of health, which predispose persons to particular health conditions, affect ability to reach and utilise health facilities, and influence the effects of services toward health. Yet, as revealed by the study data and previous research, healthcare professionals often feel helpless when faced with the complex health and social challenges of patients [58]. It is vital that HCPs are trained and supported to assess and address how social realities may be influencing a person’s health and affecting their ability to engage with advice or treatment plans [59]. A first step in addressing often hidden social issues may be asking patients about potential social challenges in a sensitive way. There are a growing number of clinical tools to help frontline practitioners ask about factors such as employment, housing or barriers to making appointments [60, 61]. Once a “social diagnosis” has been made, HCPs should be enabled to connect patients with various support resources, such as local peer-to-peer learning groups or employment agencies. Person-centred health interventions for heart failure patients must also be tailored to correct for prevalent negative social determinants [62]. 

Thirdly, this study indicated that person-centred service delivery for heart failure necessitates engaging patients and families in managing their care. This requires providing patients and caregivers with information to support self-management, including how to recognise symptoms and take appropriate action. The need to enable self-management has been highlighted in heart failure care guidelines worldwide, including the Thai Cardiology Society guideline, and in prior research on heart failure in Thailand [63, 64]. This study particularly stresses the need to provide, and support the use of, monitoring and therapeutic equipment at home, and to ensure the patient’s informal caregiver(s) or family members are also provided with information to support self-management. Prioritising symptom management has the potential to improve individuals’ quality of life, whilst significantly reducing hospital readmissions and costs of care [65–67].

Regarding the health workforce, the findings underline the need for healthcare facilities to create supportive work environments for HCPs, which encourage effective, harmonious team working, and support professionals’ psychological and emotional wellbeing. This may involve multidisciplinary trainings, opportunities for HCPs to debrief with colleagues, and providing staff support services. The findings also highlight the critical importance of HCPs’ communication skills for PCC, including the ability to communicate with sensitivity, non-judgementalism and compassion, and to share tailored, easily understandable information with patients and caregivers. To enable this, healthcare systems must offer continuing professional development, education and mentorship for practicing person-centred communication [68, 69].

### Strengths and limitations

To our knowledge, this study is the first exploration of how clinical services can better deliver PCC for heart failure patients in Thailand. Collaboration across interdisciplinary researchers also allowed a range of perspectives to inform the data collection, analysis and interpretation. We acknowledge that the interviewer being a practicing psychiatrist may initially have resulted in a more clinical line of questioning and interpretive lens during the interviews, with participants also holding particular views about the interviewer’s expectations of their responses. However, the interviewer was trained and proficient in qualitative interviewing methods, was careful to stress the purpose of the research to participants, and used the reflexivity log to note any reflections on his own interviewing style, potential biases and key takeaways and discussed these regularly with another researcher (AG). We also acknowledge that the study site was a specialised clinic based in a tertiary academic hospital in an urban location. Further depth and applicability of the findings may have been achieved by inclusion of non-specialised study sites and sites in rural settings. Despite this, the study site did serve diverse populations from a range of socioeconomic brackets and geographic locations.

## Conclusion

The study findings indicate specific practice actions that contribute towards delivering PCC for persons living with heart failure in Thailand. Such actions include (i) compassionate and respectful communication by HCPs; ii) effective and harmonious teamwork amongst multidisciplinary HCPs; iii) proactive responses to physical, psychological, social, relational and information needs of patients and caregivers; iv) engaging patients and families in symptom management; v) providing opportunities for patients to be cared for in the community and at home; and vi) being responsive to the social determinants of health, illness and healthcare access. Delivering PCC in such a way could help contribute in efforts to improve outcomes for patients, enhance patients’ sense of agency and experiences of care, improve health equity, and reduce hospital admissions and health system inefficiencies.

## Supplementary Information

Supplementary Material 1.

Supplementary Material 2.

Supplementary Material 3.

Supplementary Material 4.

Supplementary Material 5.

Supplementary Material 6.

## Data Availability

Data supporting the results is provided within the manuscript.
