# Supplementary material for: How to deliver person-centred care for people living with heart failure: a multi stakeholder interview study with patients, caregivers and healthcare professionals in Thailand
Source: BMC Health Serv Res. 2024 Dec 18;24:1570. doi: 10.1186/s12913-024-11922-z (PMC11654141; doi:10.1186/s12913-024-11922-z)
Supplement: Supplementary file 4 — Supplementary Material 4. [file 12913_2024_11922_MOESM4_ESM.docx]

### Supplementary Material 4. Transcription and translation process details

All interviews were transcribed by the bilingual field researcher who conducted the interviews, and then n=18 were translated by a bilingual colleague of the interviewer and n=18 were translated by an external service. Before data collection began at each country site, the researcher led a training workshop for all local researchers, which included transcription and translation processes.

All external translators were selected first on the basis of previous experience of interview translation and secondly on the basis of the quality of one translation they provided. All professional translators signed a confidentiality agreement before translating any audio files, to protect the identity of the participants’ involved. Once translators transcribed and translated one file, the quality was checked and assessed by the field researcher who conducted the interviews. Once it was verified that the transcripts were of high quality and accuracy, the remaining interviews were first transcribed verbatim, in the original language, and then translated into English.

The translators and the field researcher were given guidelines to follow when transcribing the documents. These included transcribing verbatim, including pauses and non-verbal communication such as laughing, highlighting sections which were unclear in the recording and requesting clarification and pseudonymising the data. On completion, a random passage of each transcript and translation was checked by the field researcher who conducted the interview against the original audio recording, for accuracy and fidelity.
