# Supplementary material for: How to deliver person-centred care for people living with heart failure: a multi stakeholder interview study with patients, caregivers and healthcare professionals in Thailand
Source: BMC Health Serv Res. 2024 Dec 18;24:1570. doi: 10.1186/s12913-024-11922-z (PMC11654141; doi:10.1186/s12913-024-11922-z)
Supplement: Supplementary file 5 — Supplementary Material 5. [file 12913_2024_11922_MOESM5_ESM.docx]

### Supplementary Material 5. Reflexivity Log

**Interviewer reflection form**

| **Subject ID number:**  ________  **Interviewer Initials:** ________  **Participant sub-group type** (*circle*):  patient/carer  **Site:** _______________                **Audio file number:**  __________  **Interview Date:** ___/___/___  **Today’s date:**  ___/___/___ |
| --- |
| 1. How would you describe the atmosphere and context of the interview? 2. What were the main points made by the respondent during this interview? 3. What new information did you gain through this interview compared to previous interviews? 4. Was there anything surprising to you personally? Or that made you think differently? 5. Were there any problems with the topic guide (e.g. wording, order of topics, missing topics) you experienced in this interview? 6. Were there points where you could have asked for more information? 7. Were there points when you could have left longer for the interviewee to respond? 8. How would you reflect upon your interviewing style? Do you feel that any of your questions may have appeared judgemental, leading or insensitive? Were any of your questions particularly good at eliciting an informative response? |
