# Supplementary material for: How to deliver person-centred care for people living with heart failure: a multi stakeholder interview study with patients, caregivers and healthcare professionals in Thailand
Source: BMC Health Serv Res. 2024 Dec 18;24:1570. doi: 10.1186/s12913-024-11922-z (PMC11654141; doi:10.1186/s12913-024-11922-z)
Supplement: Supplementary file 6 — Supplementary Material 6. [file 12913_2024_11922_MOESM6_ESM.docx]

# Supplementary Material 6. Construction of the thematic coding framework

The thematic coding frame was constructed collaboratively, drawing on the local researcher’s knowledge and views throughout. First, the lead researcher in Bangkok (PP) participated in a qualitative data analysis workshop led by AG. PP and AG then both individually coded a sub-selection of the interview transcripts: three patient, three caregiver and three HCP interviews. The interviews selected were conducted at the beginning, middle, and end of the data collection time period to capture the interviewer’s evolving interviewing style and focus. PP and AG then reconvened to compare coding and develop a coding frame. The coding frame that developed comprised: preselected a priori codes consisting of Santana et al PCC model domains [1], a priori codes derived from the results of a previously conducted systematic review[2], and inductive codes derived by content-related open coding. AG then coded all the remaining transcripts using the agreed coding frame. AG indexed and sorted all interview transcripts, created a framework matrix for each broad coding frame category, and led mapping and interpretation of the data. Key findings were mapped into a framework of PCC and organised by WHO building blocks for strengthening health systems[3].

References

1. Santana, M.J., et al., *How to practice person-centred care: A conceptual framework.* Health expectations : an international journal of public participation in health care and health policy, 2018. **21**(2): p. 429-440.

2. Giusti, A., et al., *The empirical evidence underpinning the concept and practice of person-centred care for serious illness: a systematic review.* BMJ Global Health, 2020. **5**(12): p. e003330.

3. World Health Organization, *Monitoring the building blocks of health systems: a handbook of indicators and their measurement strategies.*2010, Geneva: WHO: Available at: [www.who.int/healthinfo/systems/](file:///C:\Users\ag2248\Downloads\www.who.int\healthinfo\systems\) WHO_MBHSS_2010_full_web.pdf Accessed on 03 December 2021.
